# Mitochondrial function remains impaired in the hypertrophied right ventricle of pulmonary hypertensive rats following short duration metoprolol treatment

**DOI:** 10.1371/journal.pone.0214740

**Published:** 2019-04-09

**Authors:** Amelia S. Power, Ruth Norman, Timothy L. M. Jones, Anthony J. Hickey, Marie-Louise Ward

**Affiliations:** 1 Department of Physiology, School of Medical Sciences, University of Auckland, Auckland, New Zealand; 2 School of Biological Sciences, University of Leeds, Leeds, United Kingdom; 3 School of Biological Sciences, University of Auckland, Auckland, New Zealand; University of Canberra, AUSTRALIA

## Abstract

Pulmonary hypertension (PH) increases the work of the right ventricle (RV) and causes right-sided heart failure. This study examined RV mitochondrial function and ADP transfer in PH animals advancing to right heart failure, and investigated a potential therapy with the specific β_1_-adrenergic-blocker metoprolol. Adult Wistar rats (317 ± 4 g) were injected either with monocrotaline (MCT, 60 mg kg^-1^) to induce PH, or with an equivalent volume of saline for controls (CON). At three weeks post-injection the MCT rats began oral metoprolol (10 mg kg^-1^ day^-1-^) or placebo treatment until heart failure was observed in the MCT group. Mitochondrial function was then measured using high-resolution respirometry from permeabilised RV fibres. Relative to controls, MCT animals had impaired mitochondrial function but maintained coupling between myofibrillar ATPases and mitochondria, despite an increase in ADP diffusion distances. Cardiomyocytes from the RV of MCT rats were enlarged, primarily due to an increase in myofibrillar protein. The ratio of mitochondria per myofilament area was decreased in both MCT groups (p ≤ 0.05) in comparison to control (CON: 1.03 ± 0.04; MCT: 0.74 ± 0.04; MCT + BB: 0.74 ± 0.03). This not only implicates impaired energy production in PH, but also increases the diffusion distance for metabolites within the MCT cardiomyocytes, adding an additional hindrance to energy supply. Together, these changes may limit energy supply in MCT rat hearts, particularly at high cardiac workloads. Metoprolol treatment did not delay the onset of heart failure symptoms, improve mitochondrial function, or regress RV hypertrophy.

## Introduction

Pulmonary hypertension (PH) results in right ventricular (RV) hypertrophy and subsequent right-sided heart failure. The progression of RV hypertrophy to failure in monocrotaline-induced PH is correlated with mitochondrial dysfunction [1]. Mitochondria are vital for providing the heart with the energy for contraction and relaxation, supplying up to 95% of the ATP via oxidative phosphorylation. Previous studies have shown impaired mitochondrial complex I-fuelled respiration and variable changes in complex II-fuelled respiration in the RV of rats with PH [1–3]. These studies consistently reported that RV contractile function was significantly impaired, particularly at increased workloads, when energy demands are highest. Structural remodelling of cardiomyocytes that occurs with development of RV hypertrophy may further impair ADP channelling between the myofibrils and mitochondria, as observed in failing spontaneously hypertensive rat (SHR) hearts [4]. Impaired transfer of ADP (channelling) in SHR hearts was associated with an increase in mitochondrial reactive oxygen species (ROS) [4]. Increased mitochondrial ROS production above capacities of oxidative defence systems is self-damaging, since the ROS producing complexes (namely CI and CIII) of the electron transfer system (ETS) are also prime targets for oxidative damage [5, 6].

Increased sympathetic nerve activity (SNA) to the heart is characteristic of heart failure, and is associated with maladaptive cardiac remodelling. Increased SNA is observed in the form of increased plasma noradrenaline levels and elevated sympathetic nerve activity within skeletal muscles in PH patients [7, 8]. Although it has been questioned whether these levels reach clinical significance, a correlation between increased sympathetic nerve activity and clinical deterioration of patients has been observed [9]. β-adrenergic receptor (AR) blockade forms the basis for successful treatment of left heart failure [10–14]. However, the use of β-AR blockers in PH patients is thought to be unsafe due to the reliance of the right ventricular output on heart rate and the negative chronotropic effects of β-AR blockers [15]. Despite this, observational trials of PH patients who receive β-AR blockers for co-morbidity reasons demonstrated the safety of the drugs in this cohort [16, 17]. Promising preclinical experimental evidence for β-AR blocker treatment for right heart failure in rat models of PH is now emerging [18–22].

We recently demonstrated that the typical positive inotropic response from β-AR stimulation is absent in isolated RV tissue from rats with PH [23]. In addition, β-AR stimulation initiated significant increases in diastolic Ca^2+^, which would be expected to increase energetic demand due to futile Ca^2+^ cycling [23]. Although treatment of heart failure with β-AR blockers is well established [24], we are not aware of any studies investigating mitochondrial energy dynamics in β-blocker treated right heart failure.

In the right and left ventricles, β_1_ and β_2_-ARs are expressed in a 70:30 ratio [25, 26]. Both are coupled to the G_s_ subtype of G-protein coupled receptors (GPCRs) which initiates the cAMP/PKA signalling pathway [27]. Therefore, acute stimulation of β_1_-ARs increases the energy demands of the heart. Chronic stimulation of β_1_-ARs is associated with harmful processes including cell death, fibrosis and remodelling [28]. However, stimulation of β_2_-ARs, which can also couple to the G_i_ subtype, may actually be protective [29, 30]. Metoprolol is a second generation selective β_1_-AR blocker, and has been shown to improve survival in rats with monocrotaline-induced PH, resulting in decreased RV hypertrophy and improved RV function assessed by echocardiography [22]. Metoprolol was chosen to specifically treat the failing myocardium, as it has no action on α-AR and β_2_-AR and no direct effects on the pulmonary vasculature as seen with e.g. carvedilol [18, 20]. Thus the action of metoprolol is distinct from current treatment strategies that act to decrease afterload on the RV [15].

Attenuating β-AR mediated signal transduction (elevated cAMP and PKA activity) is considered to be the main beneficial effect of β_1_- and β_2_-AR blocker treatment, but additional benefits have been found to improve sub-cellular remodelling and oxidative stress (for review see [14]). Metoprolol treatment in chronic LV heart failure can improve systolic function and cause regression of hypertrophy on organ [31, 32] and cardiomyocyte levels [33]. It is unclear whether blocking β_1_-AR can improve myocardial energy metabolism: it has been shown both to restore the [CrP]/[ATP] ratio [34, 35], or to have no effect [36] in animal models of heart failure. Additionally, in a rat model of heart failure, treatment with the β_1_-AR blocker bisoprolol post-myocardial infarction increases cytosolic and mitochondrial creatine kinase (CK) activities, which should act to improve ATP transfer and buffering within cardiomyocytes [35].

Cardiac function is reliant on mitochondrial ATP for energy supply which is increased during β-adrenergic stimulation. The use of β-blockers might therefore be expected to reduce energy demands on the heart. However, it is not known if β-blocker treatment with metoprolol can also improve mitochondrial function and energy transfer in right heart failure. This study aims to test two hypotheses. (1) That right heart failure induced by PH contributes to an impairment of mitochondrial function and effective ADP channelling (transfer) between the myofibrils and mitochondria, and this increases ROS production. (2) That the β_1_-AR selective blocker metoprolol treatment can regress right heart failure, improve mitochondrial ADP channelling, and decrease mitochondrial ROS production. To test these hypotheses, we studied the monocrotaline (MCT) rat model of PH and right heart failure, along with their age-matched healthy controls.

## Methods

### Animal model and metoprolol treatment

PH and RV hypertrophy was induced in male Wistar rats (317 ± 4 g; bred at the University of Auckland with breeding pairs originally from Charles River Laboratories, USA) by a single intraperitoneal injection of 60 mg kg^-1^ monocrotaline in saline (MCT; n = 12). Controls were injected with an equivalent volume of saline (CON; n = 6). Rats were fed normal rat chow and water *ad libitum* for up to six weeks. Rats were monitored (weight gain, water intake and food consumption) twice weekly for the first two weeks post-injection and then daily once metoprolol dosing began. Two days before commencing metoprolol dosing, the rats were trained to voluntarily ingest sucrose solution (20% (v/v) Ribena and 10% (w/v) sucrose in water) administered by hand from a 1 ml needleless syringe. At day 20 post-MCT injection, dosing of rats with metoprolol commenced. Metoprolol tartrate (1.25 mg mL^-1^; Santa Cruz Biotechnology, USA) was dissolved in the sucrose solution and administered (10 mg kg^-1^ day^-1^) to the treatment group of MCT rats (MCT + BB; n = 6) half an hour before their dark cycle. The dose of metoprolol used was equivalent to that used by Fowler et al. (2018) [21], but in the present study dosing commenced at a later stage of development as older animals were used; these demonstrate a longer time to reach end-stage heart failure based on previous work by our colleagues [37]. Remaining MCT and control rats were fed an equivalent volume of the sucrose solution. In the fourth to fifth week post-injection the MCT and MCT + BB rats were monitored closely for signs of heart failure. Overt signs of heart failure included rats being non-inquisitive, having hunched posture, lack of grooming, dyspnea, cold/pale extremities, pilo-erected fur, porphyrin around the eyes and nose, and significant weight loss. Humane end-points were determined as weight loss of more than: (1) 15% of body weight in 24 hours; (2) 20% of body weight or more plus one other clinical (overt) sign compared with control; or (3) 25% compared with control. All untreated MCT rats were sacrificed when signs of heart failure were observed, and MCT + BB rats were sacrificed at a comparable time point. The use of animals for this study was approved by the University of Auckland Animal Ethics Committee (reference 001403).

On the day of experimentation, animals were weighed, anaesthetised with isoflurane and decapitated before excising the heart. The heart was immediately rinsed in ice-cold Tyrode’s solution prior to blotting and weighing. The wet and dry weights of the liver and lungs were obtained, as well as the tibial length measurement. During the RV tissue dissection, hearts were Langendorff-perfused with oxygenated Tyrode’s solution containing (in mM): NaCl (141.8), KCl (6), MgSO_4_.H_2_O (1.2), Na_2_HPO_4_ (1.2), 10 HEPES (10), glucose (10), CaCl_2_ (0.25) and 2,3-butanedione monoxime (20) at pH 7.4. Approximately 50 mg of tissue was dissected from the apex of the RV free wall and stored in ice-cold permeabilisation buffer containing (in mM): CaK_2_EGTA (2.77; 0.1 μM free Ca^2+^), imidazole (20), taurine (20), K-MES (4-morpholineethanesulfonic acid; 50), dithiothreitol (0.5), MgCl_2_ (6.56), ATP (5.77), phosphocreatine (15) at pH 7.1. Another two samples of the free wall were removed. One sample was snap frozen in liquid nitrogen and stored at -80 °C for determination of citrate synthase enzyme activity and soluble protein content. The other sample was fixed in 1% paraformaldehyde for confocal imaging. Measurements of the left and right ventricle free wall thickness were obtained using micro-callipers.

### Measurement of mitochondrial respiration in permeabilised muscle fibres

Two assays were performed to assess both the maximum oxidative phosphorylation (OXPHOS) capacity of the mitochondria *in situ* (assay 1), as well as the connectivity between the cytosolic ATPases and the mitochondria within cardiomyocytes (assay 2) [4]. Assays were performed using an Oroboros-O2k oxygraph (Oroboros Instruments, Austria) with each chamber equilibrated at 37 °C with 2 mL of respiration buffer containing (in mM): MgCl_2_.6H_2_O (3), K-lactobionate (60), taurine (20), KH_2_PO_4_ (10), HEPES (20) and sucrose (110), with essential fatty acid free bovine serum albumin (BSA; 1 g L^-1^) at pH 7.1. RV tissue was saponin-permeabilised, as detailed previously [38]. O_2_ concentration in the chambers was kept above 350 μM in order to overcome O_2_ diffusion barriers in the fibres. Steady-state respiration rate was achieved before the titration of each substrate into the chambers.

#### Assay 1: Exogenously stimulated oxidative phosphorylation

Mitochondrial respiration was measured from permeabilised fibres in the presence of complex I (CI) substrates glutamate (10 mM), malate (5 mM) and pyruvate (10 mM), in the absence of ADP. This is known as the “Leak” state (NADH-stimulated Leak). Maximum CI OXPHOS was stimulated with saturating MgADP (2.5 mM) [39] followed by the addition of complex II (CII) substrate succinate (10 mM) for combined CI and CII OXPHOS. OXPHOS stimulated with saturating exogenously added ADP is referred to as OXPHOS.

#### Assay 2: Endogenously stimulated oxidative phosphorylation with simultaneous measurement of reactive oxygen species

**Fig 1:**
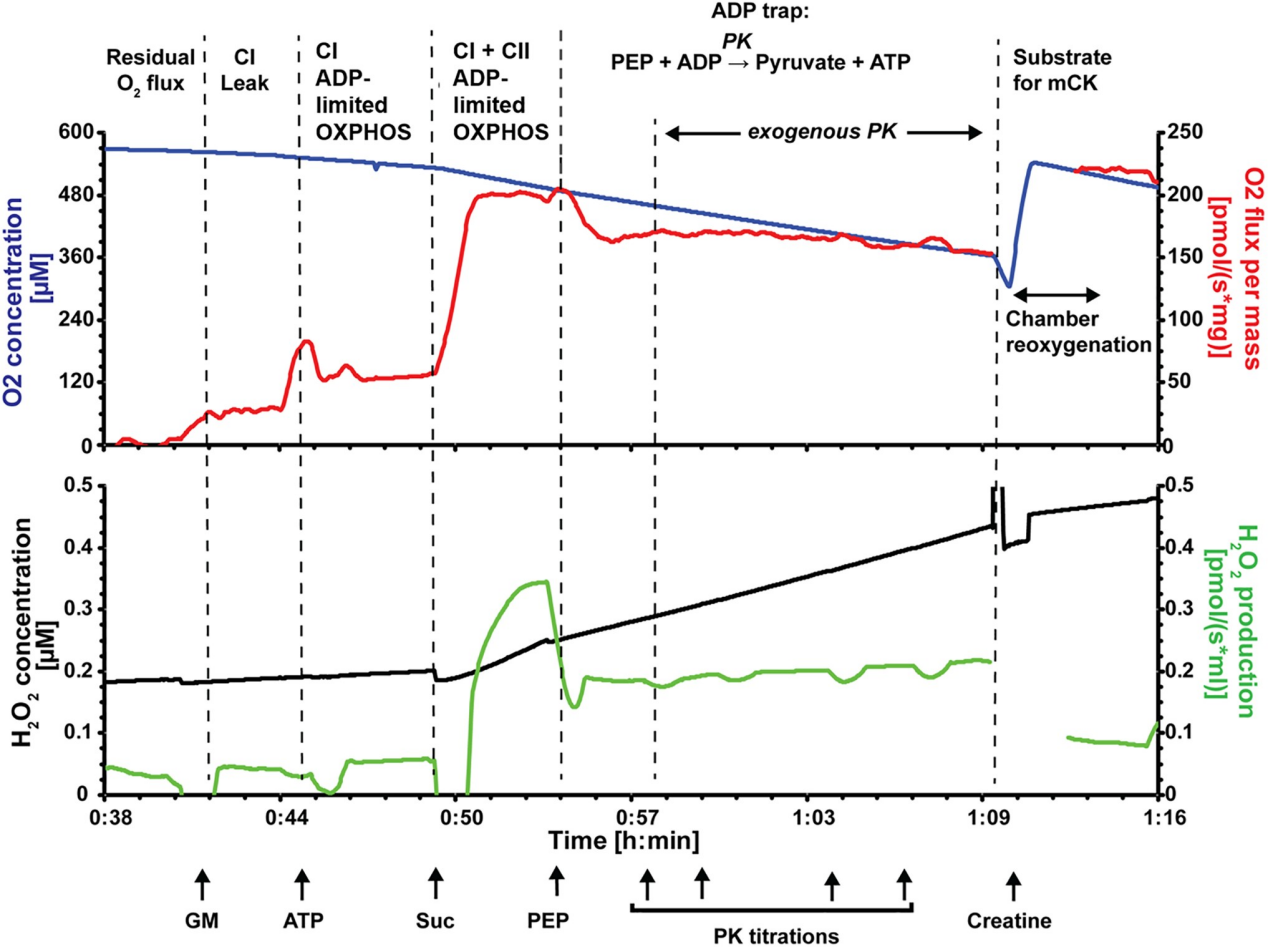
Representative oxygraph trace measuring endogenous OXPHOS and ADP channelling from myofibrils to the mitochondria within permeabilised fibres. The upper trace shows the O_2_ concentration (μM; blue) and mass specific O_2_ flux (pmols s^-1^ mg^-1^; red). The lower trace shows concurrent fluorescent measurement of total ROS (O^-^_2_. + H_2_O_2_) with enzyme coupled detection system Amplex UltraRed, super oxide dismutase and horse radish peroxidase. Step wise addition of substrates allows measurement of different respiratory states. Glutamate and malate (GM) produced NADH-stimulated Leak (CI Leak); ATP stimulates oxidative phosphorylation (OXPHOS) respiration through endogenous production of ADP by cellular ATPases (CI ADP-limited OXPHOS); succinate is a complex II substrate (CI + CII ADP-limited OXHOS); phosphoenolpyruvate (PEP) is a substrate for endogenous pyruvate kinase (PK) and a reaction that consumes ADP (ADP trap); additional titration of exogenous PK further tests ADP channelling towards mitochondria. Finally, addition of creatine stimulates mitochondrial creatine kinase (mCK), promoting local turnover of ADP within the intermembrane space and relieving inhibition of the ADP trapping enzyme system. Artefacts in the traces (red and green) during the addition of substrates or O_2_ have been omitted for clarity.

Mitochondrial respiration was measured simultaneously with ROS production (Fig 1). ROS were monitored using fluorescent sensors to detect Amplex UltraRed (AMPR; ThermoFisher Scientific) reagent product Amplex UltroxRed [40]. Approximately 2 mg of permeabilised fibres were added to each oxygraph chamber containing respiration buffer supplemented with AMPR (5 μM), superoxide dismutase (SOD; 5 U mL^-1^), and horse radish peroxidase (HRP; 10 U mL^-1^). In the presence of SOD, superoxide (O^-^_2_.) is converted to H_2_O_2_. The H_2_O_2_ produced, along with endogenously produced H_2_O_2_, reacts with AMPR in the presence of HRP to produce the fluorescent product (Amplex UltroxRed) that represents total ROS production. The signal is calibrated with H_2_O_2_ (330 nM) in the chamber before the addition of permeabilised fibres. CI substrates glutamate (10 mM) and malate (5 mM), were added to induce NADH-stimulated Leak. MgATP (2.5 mM) was then added to measure OXPHOS stimulated by endogenously produced ADP (referred to as ADP-limited OXPHOS), followed by the addition of CII substrate succinate (10 mM). Phosphoenolpyruvate (PEP) was then added as an ‘ADP trap’ to stimulate endogenous ADP scavenger, pyruvate kinase (PK, Eq 1). Exogenous PK was then titrated (1 U, 1 U, 2 U, 2 U) into the chamber to further scavenge ADP and test the connectivity between the myofibrils and mitochondria [4]. Pyruvate kinase competes with the mitochondria for ADP, therefore, if there is a short distance between the cytosolic ATPases and mitochondria, then there will be less inhibiton of respiration with PEP + PK.

$$ADP+PEP\;\overset{PK}{\to }\;ATP+pyruvate$$ (1)

Creatine (10 mM) was then added to the chamber to stimulate localised production of ADP by mitochondrial creatine kinase within the mitochondrial inter membrane space, which cannot be accessed by ADP scavenger PK [41]. Following creatine, FCCP was added to uncouple the mitochondria and measure maximum ETS capacity (ET). Finally, antimycin A (25 μM) was added to determine background O_2_ consumption.

### Citrate synthase activity and soluble protein content

Citrate synthase and soluble protein content was measured from the frozen RV samples using colourmetric 96-well plate based assays measured with a Spectramax 340PC plate reader (Molecular Devices, USA). Tissue was weighed (~20 mg) and homogenised 1:20 (w/v) in homogenization buffer containing (in mM) EDTA (1), MgCl_2_ (2), KCl (50), Tris (25) and 0.1% Triton X-100 at pH 7.8. The homogenate was centrifuged at 17,000 x g for 10 minutes. The supernatant was collected and diluted 5 fold before 5 μL was added to 180 μL of reaction buffer containing (in mM) acetyl coenzyme A lithium salt (0.1), (5,5’-dithiobis (2-bitrobenzoic acid) (DTNB; 0.2) and Tris (50) at pH 8.0 in a 96 well plate. The reaction was then started by the addition of oxaloacetate (0.5 mM) and the production of TNB was followed at 412 nm [42]. The enzyme activity was normalised to soluble protein content measured using the bicinchoninic acid method [43].

### Immunolabelling and confocal imaging and analysis

Three hearts from each rat group were used for imaging and analysis. RV tissue blocks were immersion-fixed in 1% paraformaldehyde for 1 hour at 4 °C before being cryo-protected with 10, 20 then 30% sucrose in phosphate buffered saline (PBS). The blocks were then frozen in liquid nitrogen, cooled in 2-methylbutane and cryosectioned into 20 μm transversely orientated sections. Sections were incubated for 1 hour with image iT FX signal enhancer (Life Technologies) and washed once with PBS for 5 min. Sections were triple labelled for mitochondria (Tom20), *f*-actin (phalloidin) and extracellular matrix (wheat germ agglutinin, WGA) as previously described [4]. The sections were incubated overnight at 4 °C with primary antibody rabbit anti-Tom20 (1:200, sc-11415, Santa Cruz Biotechnology). Sections were washed before 2 hours of incubation at room temperature with secondary goat anti-rabbit Alexa 488 (1:200, Thermofisher Scientific) Alexa 594-phalloidin (1:50, Thermofisher Scientific) and Alexa 680-WGA (1:50, Thermofisher Scientific). Sections were washed and mounted in Prolong Gold (Thermofisher Scientific). Three-dimensional images (0.25 μm spacing, 82 nm pixel size) were acquired with a Zeiss 710 laser scanning microscope using a Zeiss 63x oil-immersion objective NA 1.40. Images were deconvolved using the Richardson-Lucy algorithm described by Soeller and Cannell (1999) [44]. The cross-sectional area of the cardiomyocytes was determined from the WGA label, and then the densities of mitochondria and f-actin were measured by creating a mask for each in Fiji (imageJ). The average distance of each pixel to the edge of the labelled area (mitochondrial clusters or myofibrils) was then calculated using a skeletonization and edge-detection script in Python (Version 7.3.2) [4]. Fibrosis was quantified from the WGA labelling of the extracellular matrix which has been shown previously to be a suitable method for detection and quantification of fibrosis in cardiac tissue [45].

### Data analysis

Measurements of O_2_ consumption rates and ROS production were recorded in DatLab6 (Oroboros Instruments, Austria) for each RV sample. Averaged steady-state data was exported, and is presented as mean ± SEM for the different respiration states. To infer CI activity, the rate of NADH-stimulated OXPHOS was normalised to OXPHOS with succinate (CI OXPHOS/ (CI + CII OXPHOS)).

### Statistics

Statistical analysis (MCT vs CON vs MCT + BB) was determined by one-way analysis of variance (ANOVA) followed by Sidak’s multiple comparisons test using Prism 7 (GraphPad Software, USA). Differences in ROS production between respiratory states were tested with a paired t-test.

### Chemicals and solutions

Unless otherwise specified, all reagents and chemicals were purchased from Sigma-Aldrich (USA).

## Results

### Animal growth and morphometric data

**Fig 2:**
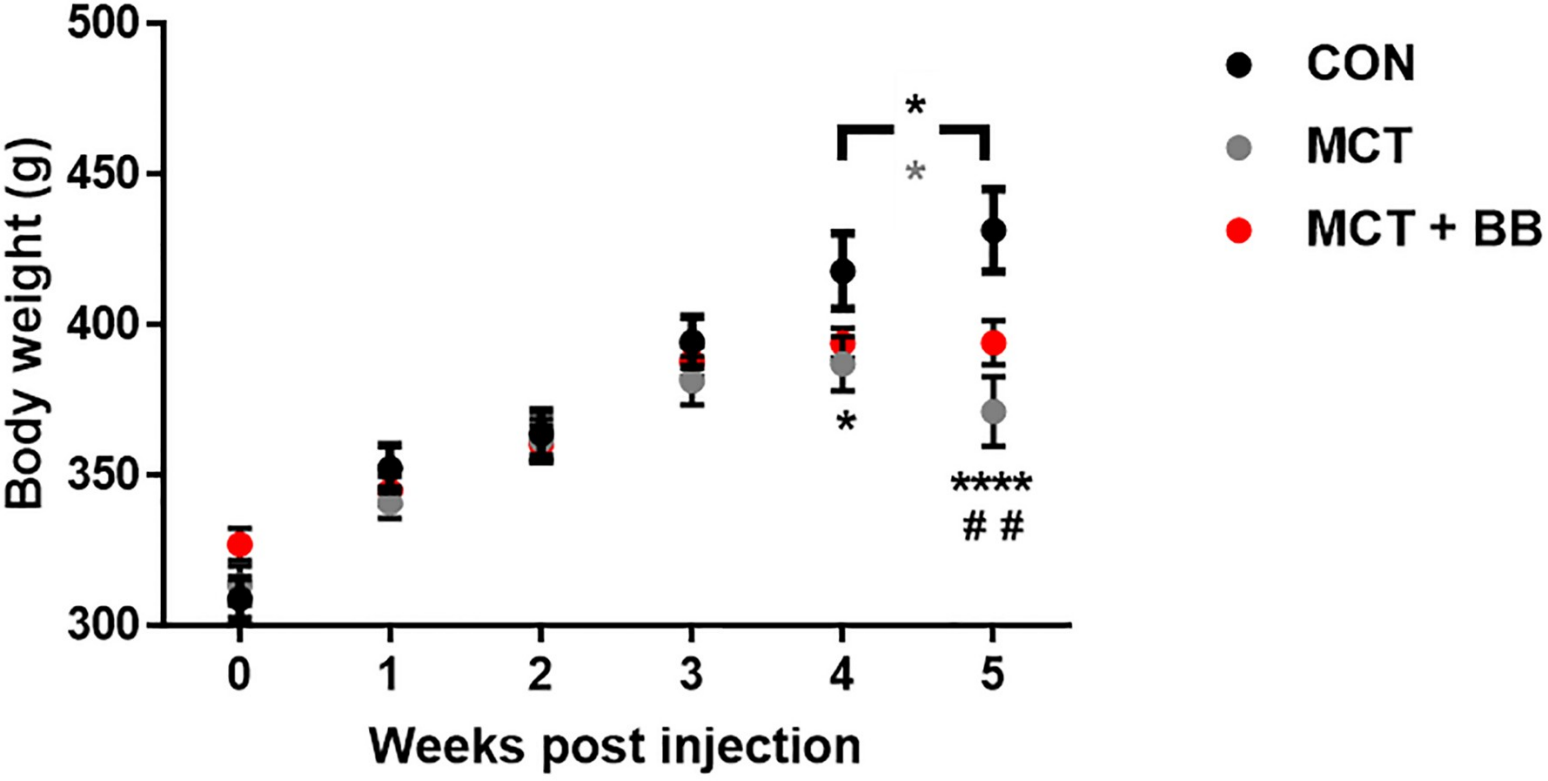
Growth trajectory following injection. Mean ± SEM body weight of rats following injection of saline (CON; black; n = 6), monocrotaline (MCT; grey; n = 5) or monocrotaline plus metoprolol treatment (MCT + BB; red; n = 6). Statistical significance between CON vs. MCT (*) or CON vs. MCT + BB (^#^) are denoted by: * p < 0.05; ** or ^##^ p < 0.01; **** p < 0.0001; using two-way ANOVA with multiple comparisons and matching. Significant differences between weeks 4 and 5 are denoted by a black * for CON, and a grey * for MCT.

Fig 2 shows the growth trajectory for rats post-monocrotaline or -saline injection. By the fifth week post-injection, both MCT and MCT + BB rats were significantly lighter than controls and displayed clinical signs of heart failure. However, while there was a significant drop in body weight in the MCT rats, and a gain in the control rats between weeks four and five, body weight tended to plateau in the MCT + BB group. All rats injected with monocrotaline displayed overt signs of heart failure by the fifth week, including those treated with metoprolol.

Both the MCT and MCT + BB animals had trends towards greater absolute lung wet weights compared to controls (CON vs. MCT: p = 0.13; CON vs. MCT + BB: p = 0.058). Both MCT and MCT + BB rats had increased heart weight and RV hypertrophy relative to controls, with no change in the LV free wall thickness (Table 1). There was no significant difference in the liver wet weight or tibial length between groups. None of the morphometric data was effected by metoprolol treatment, other than the heart:body weight %, which was marginally lower in the MCT + BB (CON: 0.34 ± 0.01%; MCT: 0.59 ± 0.03%; MCT + BB: 0.51 ± 0.02%; p ≤ 0.05 for CON vs MCT, CON vs MCT + BB and MCT vs. MCT + BB).

**Table 1:** Morphometric data.

	CON	MCT	MCT + BB	p ≤ 0.05
Days post injection	38 ± 4	35 ± 7	32 ± 3	#
Final Body weight (g)	441 ± 12	367 ± 10	395 ± 8	* #
Heart weight (g)	1.49 ± 0.07	2.02 ± 0.17	2.00 ± 0.05	* #
Tibia length (mm)^Ɨ^	55 ± 1	54 ± 1	51 ± 2	
Heart:tibia length (g/cm) ^Ɨ^	0.27 ± 0.01	0.40 ± 0.02	0.39 ± 0.02	* #
Lung weight (g)	1.82 ± 0.11	2.41 ± 0.25	2.62 ± 0.26	
Lung:tibia length (g/cm) ^Ɨ^	0.33 ± 0.02	0.47 ± 0.05	0.53 ± 0.07	
Liver weight (g) ^Ɨ^	14.9 ± 0.3	13.0 ± 0.6	14.5 ± 1.2	
RV free wall (mm)	1.5 ± 0.1	2.7 ± 0.2	2.2 ± 0.1	* #
LV free wall (mm)	4.0 ± 0.1	3.7 ± 0.2	4.1 ± 0.22	

Measurements from control (CON; n = 6), monocrotaline (MCT; n = 6, ^Ɨ^n = 5), and monocrotaline treated with metoprolol (MCT + BB; n = 6) animals. All data is expressed as the mean ± SEM except for days post injection which is the median day ± range. Significant differences are denoted by

* p ≤ 0.05 for CON vs. MCT,

^#^ for CON vs. MCT + BB using an ordinary one-way ANOVA with Sidak’s multiple comparisons.

### Mitochondrial respiration and ROS production in permeabilised fibres

Mitochondrial respiration was measured in permeabilised fibres stimulated with CI and CII substrates in the presence of saturating exogenous MgADP (OXPHOS; assay one) or MgATP which promotes endogenous turnover of ATP to ADP by cytosolic ATPases and subsequent stimulation of OXPHOS (ADP-limited OXPHOS; assay two).

#### Assay 1: Exogenously stimulated oxidative phosphorylation

**Fig 3:**
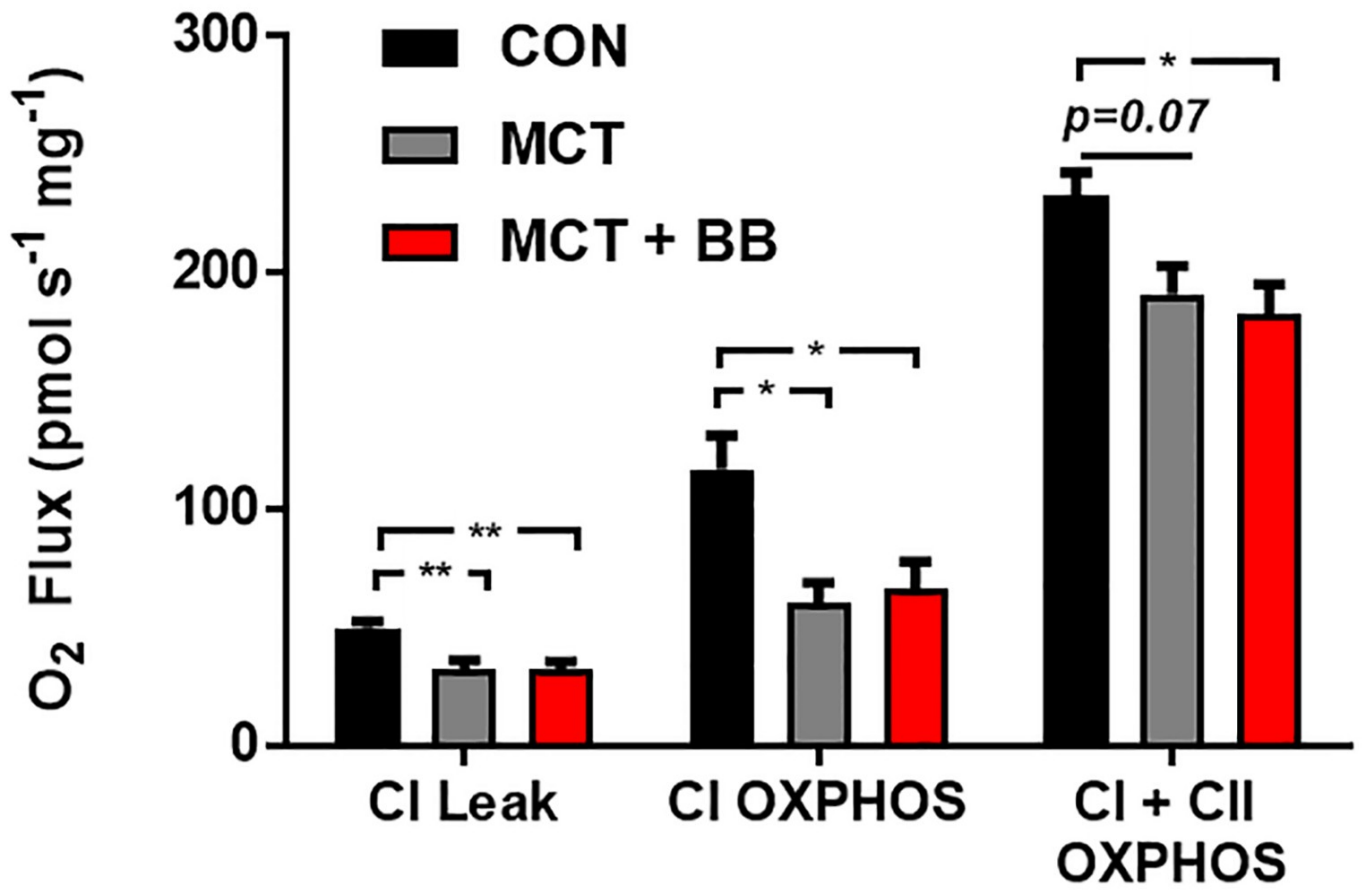
Respiration of permeabilised fibres stimulated with saturating ADP. Mean ± SEM O_2_ flux normalised to wet weight (pmol s^-1^ mg^-1^) are shown in black for the controls (CON; n = 6), in grey for monocrotaline (MCT; n = 5) and in red for metoprolol treated monocrotaline rats (MCT + BB; n = 6). Steady-state measurements were taken in the presence of complex I substrates (CI Leak for NADH-stimulated Leak), after the addition of ADP (CI OXPHOS) and finally complex II substrate succinate (CI + CII OXPHOS). Statistical significance are denoted by * p < 0.05, ** p < 0.01, using one-way ANOVA with Sidak’s multiple comparisons.

O_2_ flux was lower in both the MCT and MCT + BB groups relative to controls in the NADH-stimulated Leak state and when respiration was measured with saturating MgADP (CI OXPHOS) (Fig 3 & Table 2). Following the addition of complex II substate succinate, O_2_ flux (CI + CII OXPHOS) remained lower in the MCT + BB group, but there was only a trend towards significance in the MCT group (p = 0.07). There was no improvement seen in the MCT + BB group relative to the MCT group in the OXPHOS states. NADH-stimulated OXPHOS was normalised to CI + CI OXPHOS to infer CI activity (CI OXPHOS/(CI + CII OXPHOS). In the control fibres NADH stimulated activity accounted from approximately half of the total flux (CON: 0.51 ± 0.06), while it only accounted for a third of the total flux in the MCT and MCT + BB fibres (MCT: 0.34 ± 0.02; MCT + BB: 0.35 ± 0.03). Therefore, the contribution of CII activity to overall CI + CII OXPHOS is greater in the MCT and MCT + BB hearts.

**Table 2:** O_2_ flux of permeabilised fibres under different respiratory states.

**Assay One**	**CON**	**MCT**	**MCT + BB**	**p ≤ 0.05**
CI + CII OXPHOS (ADP) (pmols s^-1^ mg^-1^)	233 ± 10	191 ± 12	182 ± 12	* #
CI / CI + CII OXPHOS (ADP)	0.51 ± 0.06	0.34 ± 0.02	0.35 ± 0.03	* #
**Assay Two**	**CON**	**MCT**	**MCT + BB**	**p ≤ 0.05**
CI + CII ADP-limited OXPHOS (ATP) (pmols s^-1^ mg^-1^)	207 ± 9	198 ± 24	141 ± 7	# p
CI / CI + CII ADP-limited OXPHOS (ATP)	0.59 ± 0.01	0.52 ± 0.03	0.54 ± 0.02	
ET (pmols s^-1^ mg^-1^)	294 ± 18	242 ± 32	200 ± 17	#

Mean ± SEM steady-state respiratory states (pmol s^-1^ mg^-1^) from control (CON; n = 6), monocrotaline (MCT; n = 5) and metoprolol treated MCT (MCT + BB; n = 6) permeabilised RV fibres. Respiration was measured with combined CI (glutamate, malate and pyruvate) and CII (succinate) substrates stimulated with either ATP (ADP-limited OXPHOS) or ADP (OXPHOS). Fractional CI acitivity was determined by normalising CI OXPHOS O_2_ flux to combined CI + CII OXPHOS (CI / CI + CII). The maximum ETS flux (ET) was measured following uncoupling with FCCP (MCT; n = 4).

* p ≤ 0.05 for CON vs. MCT and

^#^ for CON vs. MCT + BB and Ɨ for MCT vs. MCT + BB using an ordinary one-way ANOVA with Sidak’s multiple comparisons.

#### Assay 2: Endogenously stimulated oxidative phosphorylation with simultaneous measurement of reactive oxygen species

**Fig 4:**
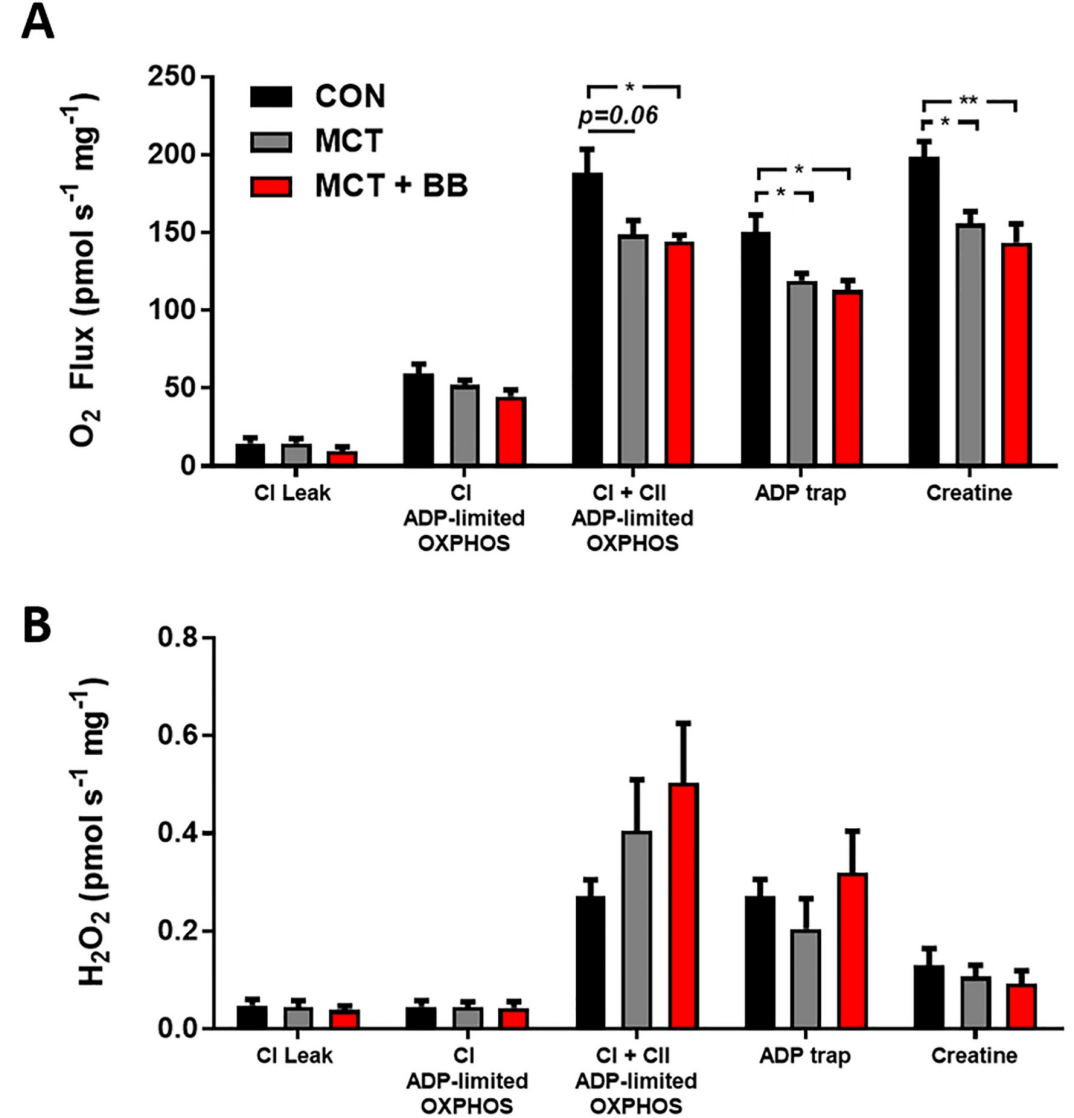
O_2_ flux and ROS production from permeabilised fibres with endogenous ADP. Mean ± SEM measurements are shown in black for the controls (CON; n = 6), in grey for monocrotaline (MCT; n = 5) and red for metoprolol treated monocrotaline rats (MCT + BB; n = 5–6). Steady-state measurements were taken during different respiration states outlined in Fig 1. NADH-stimulated Leak (CI Leak); **A: O**_2_ flux normalised to wet weight (pmol s^-1^ mg^-1^). B: Total ROS production normalised to wet weight (pmol s^-1^ mg^-1^). Statistical significance between groups are denoted by * p < 0.05, ** p < 0.01 using one-way ANOVA with Sidak’s multiple comparisons.

No difference in O_2_ flux in the NADH-stimulated Leak state or following the addition of MgATP (CI ADP-limited OXPHOS) was observed between groups (Fig 4A). Following, the addition of succinate (CI + CII ADP-limited OXPHOS), O_2_ flux was more than 20% lower in the MCT and MCT + BB groups relative to controls. The addition of the ADP trap (PEP + PK) similarly dropped O_2_ flux in all groups by ~20% (CON: 19.7 ± 1.6%; MCT: 21.3 ± 2.5%; MCT + BB: 21.9 ± 3%, p = 0.82). Addition of creatine stimulated respiration in all groups by a similar amount (CON: 35 ± 10%; MCT: 30 ± 3%; MCT + BB: 26 ± 4, p = 0.70), and respiration was still ~ 20% lower in the MCT and MCT + BB groups, relative to controls. The maximum capacity of the ETS measured with FCCP (ET) was only significantly lower in the MCT + BB group (Table 2). Taken together these results suggest that there is no detectable change in connectivity between the myofibril ATPases and the mitochondria that limited OXPHOS.

ROS production was highest for all groups following the addition of succinate (CI + CII ADP-limited OXPHOS; Figs 1 & 4B). ROS production appeared to be higher in the MCT and MCT + BB group relative to the control, however, due to large variation in the ROS measurement this was not statistically significant (p = 0.21). Following the addition of phosphoenolpyruvate and PK (ADP trap), ROS production dropped in all groups (CON: p = 0.0032; MCT: p = 0.038; MCT + BB: p = 0.0053). Addition of creatine dropped ROS production further, however, it was not significant in the MCT group (CON: p = 0.019; MCT: p = 0.105; MCT + BB: p = 0.034).

#### Soluble protein and citrate synthase activity

There was no difference between groups in the soluble protein content of the tissue (CON: 4.44 ± 0.38%, MCT: 4.75 ± 0.11%, MCT + BB: 4.64 ± 0.16%; p = 0.70), or in the citrate synthase activity normalised to soluble protein amount (CON: 0.031 ± 0.001 μmol min^-1^ mg^-1^, MCT: 0.029 ± 0.001 μmol min^-1^ mg^-1^, MCT + BB: 0.030 ± 0.003 μmol min^-1^ mg^-1^; p = 0.84). Therefore, mitochondrial content measured using the common citrate synthase marker was not different between groups.

### Cardiomyocyte cross-sectional area and mitochondrial and myofibrillar density

**Fig 5:**
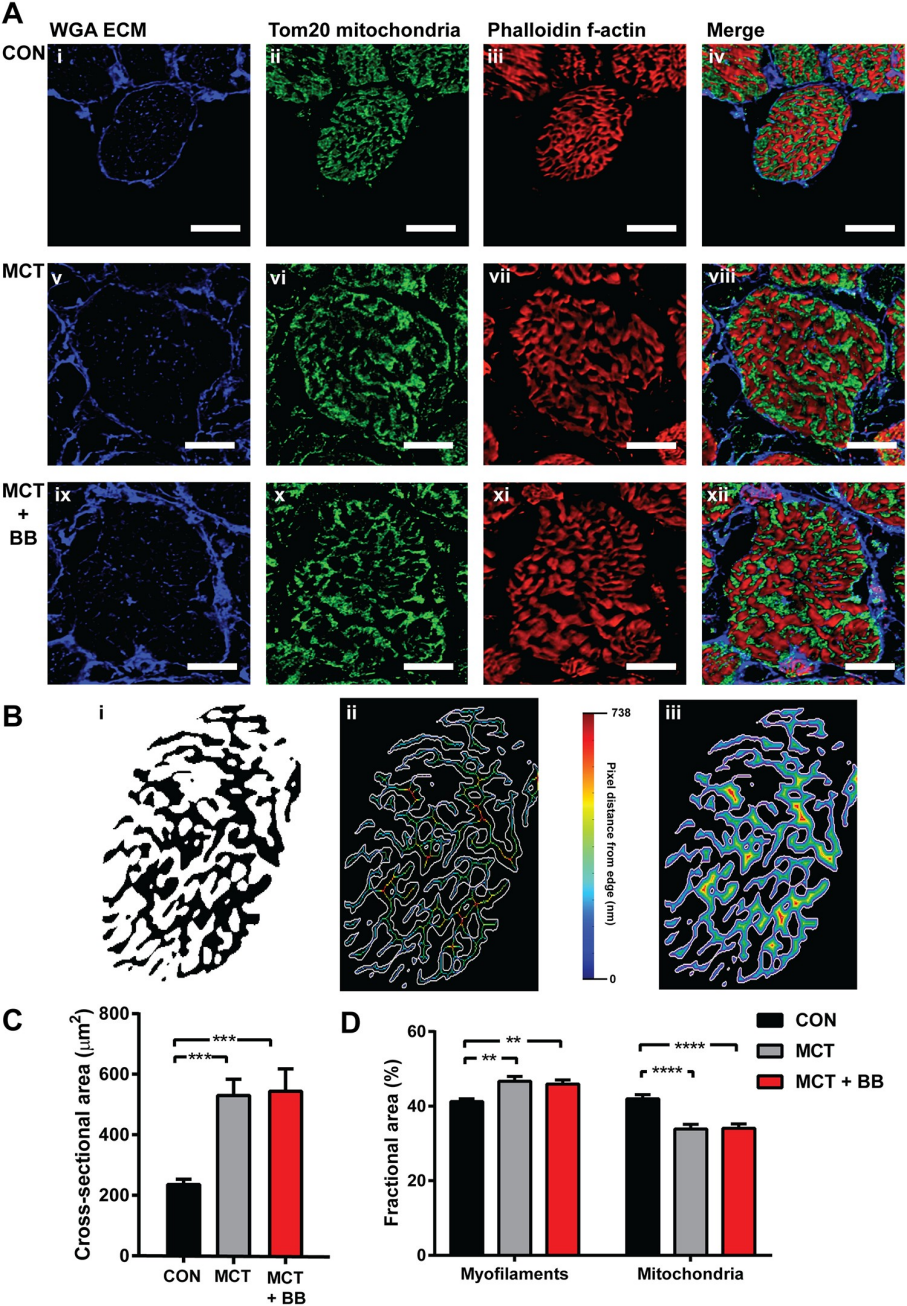
Confocal images of RV cardiomyocytes and analysis. **A:** Representative transverse sections (20 μm) labelled with extracellular matrix marker (ECM) wheat germ agglutinin (WGA) in blue (first column), mitochondrial marker anti-Tom20 in green (second row) and f-actin marker phalloidin in red (third row). The final column shows a merged image of all three channels. Scale bars are 10 μm. Images show RV cardiomyocytes from a control heart (CON; **i—iv**), a monocrotaline heart (MCT; **v—viii**) and a monocrotaline treated with metoprolol heart (MCT + BB; **ix—xii**). **B:** Mask created from image **A iii** was used to calculate fractional area of f-actin, and determine the mean distance from the skeleton (**B ii**) or from each pixel to the outer edge of the f-actin bundles (**B iii**). Visual representation is shown in images **B ii—iii** where all pixels (or skeleton pixels) are coloured according to the distance to their closest edge (white). **C:** Cross-sectional area of RV cardiomyocytes determined from tracing the WGA labelling shown in **A**. **D:** Fractional areas calculated from masks created from f-actin (myofilament) and Tom20 (mitochondria) confocal images. Control (CON; N = 3 hearts, n = 22 cells), monocrotaline (MCT; N = 3 hearts, n = 19 cells) and monocrotaline treated with metoprolol (MCT + BB; N = 3 hearts, n = 19 cells). Significant differences are denoted by ** p < 0.01, *** p < 0.001, **** p < 0.0001, using one-way ANOVA.

Confocal imaging of RV cardiomyocytes in transverse orientation confirmed cellular hypertrophy in MCT groups (Fig 5A). There was an approximate doubling of cardiomyocyte cross-sectional area with a greater variability in the MCT and MCT + BB hearts relative to controls (Fig 5C). The MCT and MCT + BB cardiomyocytes also had increased fractional areas occupied by myofilaments, and a decrease in mitochondrial fractional area relative to control cardiomyocytes (Fig 5D). The increased myofilament fractional area in the MCT groups was associated with a decrease in the perimeter/area ratio, and an increased mean pixel and to edge distance (Fig 5B & Table 3). The smaller mitochondrial fractional area did not correspond to any changes in the perimeter/area ratio, or to distances to the nearest mitochondrial edge. When expressed as a fraction of mitochondrial/myofilament area the discrepancy is highlighted in the MCT and MCT + BB groups (Table 3). Overall, in the MCT hearts, there was a 25% decrease in the fractional area occupied by mitochondria relative to myofilaments, with longer diffusion distances between the myofilaments and the mitochondria compared to control hearts.

**Table 3:** Confocal cardiomyocyte sub-cellular measurements.

	CON	MCT	MCT + BB	p ≤ 0.05
Myofilament perimeter/area	3.3 ± 0.1	2.7 ± 0.1	2.6 ± 0.1	*#
Mitochondrial perimeter/area	4.1 ± 0.1	4.5 ± 0.2	3.9 ± 0.2	
Myofilament skeleton to edge distance (nm)	300 ± 11	365 ± 15	395 ± 19	*#
Myofilament pixel to edge distance (nm)	217 ± 8	270 ± 10	291 ± 14	*#
Mitochondria skeleton to edge distance (nm)	242 ± 6	233 ± 9	253 ± 9	
Mitochondria pixel to edge distance (nm)	192 ± 5	194 ± 7	205 ± 7	
Mitochondria/myofilament area	1.03 ± 0.04	0.74 ± 0.04	0.74 ± 0.03	*#

Mean ± SEM data calculated from masks created from the f-actin (myofilament) and Tom20 (mitochondria) confocal images as described in Fig 5. Control (CON; n = 22 cells), monocrotaline (MCT; n = 19 cells) and monocrotaline treated with metoprolol (MCT + BB; n = 19 cells), 3 rat hearts per group.

* p ≤ 0.05 for CON vs. MCT and

^#^ for CON vs. MCT + BB using an ordinary one-way ANOVA with Sidak’s multiple comparisons.

### Quantification of fibrosis

**Fig 6:**
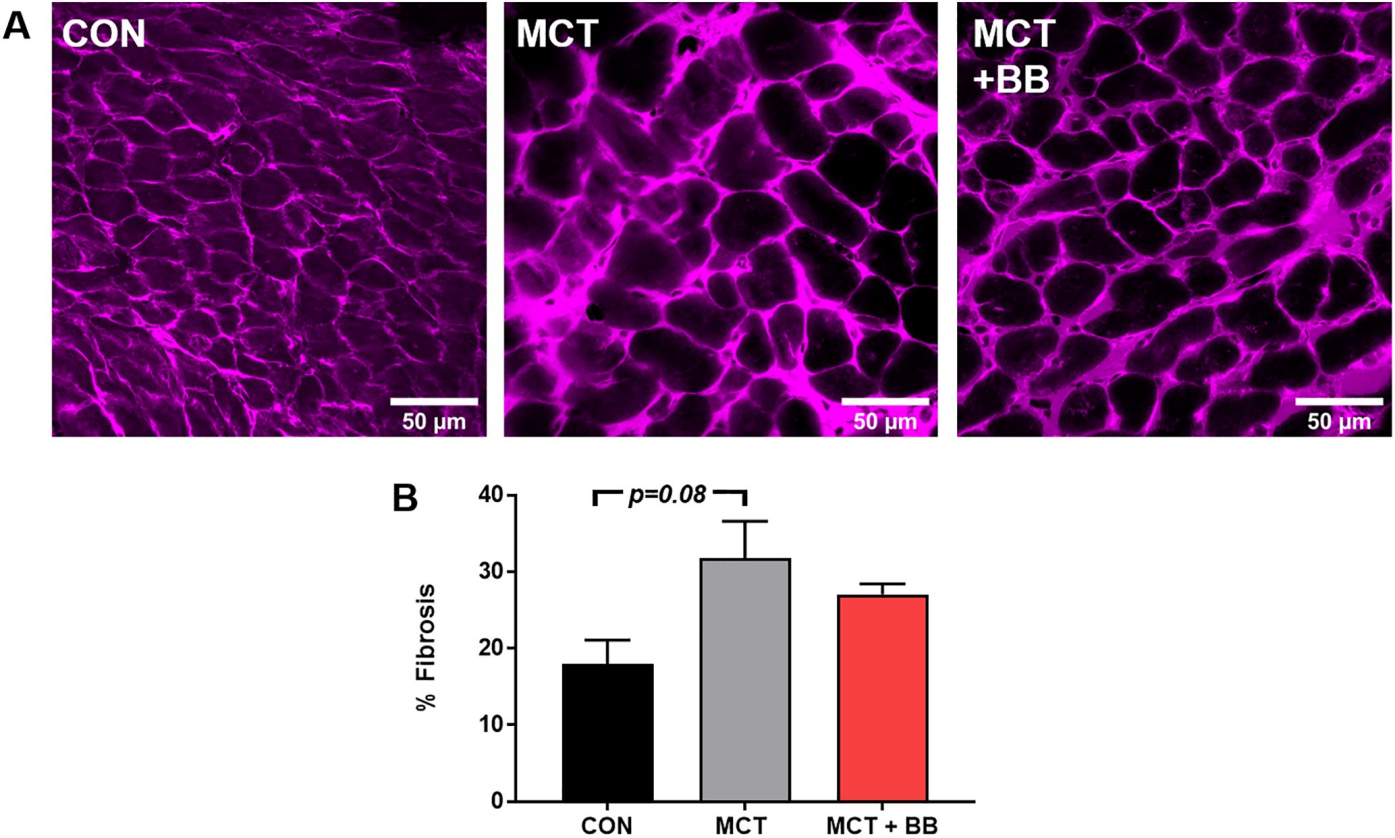
Right ventricle fibrosis. **A:** Representative overview images (248 x 248 μM) of RV tissue labelled with extracellular matrix marker, wheat germ agglutinin. Five images were taken from each heart (n = 3 hearts). **B:** Mean ± SEM from control (CON), monocrotaline (MCT) and monocrotaline treated with metoprolol (MCT + BB). A trend towards significance was found using one-way ANOVA.

Since no significant effect of metoprolol was found in the mitochondrial measures, or on RV myocyte hypertrophy, we examined the degree of fibrosis in the RV tissue as a quality control for metoprolol efficacy. There was a trend towards an increase in % fibrosis in the MCT group relative to control (CON: 17 ± 3%; MCT: 32 ± 8%; MCT + BB: 27%; p = 0.07) despite the apparent decrease in number of cells per unit area (Fig 6).

## Discussion

This study examined RV mitochondrial function and ADP channelling in MCT animals advancing to right heart failure and investigated a potential therapy with the specific β_1_-AR-blocker metoprolol. Relative to controls, MCT animals had impaired mitochondrial function but maintained coupling between myofibrillar ATPases and OXPHOS despite an increase in fractional myofibrillar content and ADP diffusion distances.

### Morphometric animal data and the onset of heart failure

Metoprolol treatment did not delay the onset of overt heart failure symptoms in MCT rats, although previous studies have shown that metoprolol delays weight loss and prolongs survival in younger animals with PH [18, 21, 22]. Han *et al*. (2017) have shown that monocrotaline injection of 300 g rats leads to a decline of heart function in the fifth week post-injection [37]. We saw significant drop in body weight in the MCT rats between weeks four to five, during the expected transition from RV hypertrophy to heart failure. Body weight tended to plateau in the MCT + BB group, suggesting some improvement in the health of the metoprolol treated rats. However, no regression of RV hypertrophy was evident in the MCT + BB rats (Table 1) and they still developed overt signs of heart failure. Rats in our study were injected at higher body weight (317 ± 4 g) than those in previous studies (~200 g) [18, 21, 22], and showed a slower progression to heart failure.

### Mitochondrial function in permeabilised RV fibres

There was no apparent benefit of metoprolol treatment in the context of mitochondrial function. Specific OXPHOS and ETS deficits were investigated by stimulating OXPHOS with the addition of saturating levels of ADP (Fig 3, and Table 2). Maximal OXPHOS O_2_ flux was depressed by ~ 50% in RV fibres from MCT and MCT + BB relative to control when provided with CI substrates, yet was only ~ 20% lower when provided with CI *and* CII substrates. This indicates that the contribution of CI to total OXPHOS is lower in MCT mitochondria at this time point. Daicho *et al*. (2009) also found a decrease in CI OXPHOS with the onset of RV heart failure (MCT induced), with no change in CII OXPHOS measured in permeabilised fibres [1]. Wust *et al*. (2016) are in agreement with our finding of depressed OXPHOS mediated by CI substrates, however they also showed depressed CII activity [2]. Taken together with the findings of our study, a decrease in O_2_ flux via CI occurs with the onset of right heart failure, while changes in CII OXPHOS flux are less conclusive. That CII mediated flux can in part compensate for decreased CI OXPHOS presents a paradox. Succinate, the CII substrate, is mostly derived from the tricarboxylic acid cycle, which is also forming NADH. NADH in excess should inhibit the tricarboxylic acid cycle, and be converted to NADPH at a cost of ATP. Succinate can also be derived from other sources (i.e. glutamate), and if so, would present a less efficient means to synthesise ATP [46].

Mitochondrial O_2_ flux was similarly 20% lower in both MCT and MCT + BB relative to controls when stimulated by endogenous ADP hydrolysed from ATP by cytosolic ATPases (Fig 4A). This requires diffusion of ADP from sites of turnover within the cardiomyocytes. The lower ADP-limited OXPHOS O_2_ flux (Fig 4A) could result from the longer diffusion distances between myofibrils and mitochondria (Table 3) in addition to the lower oxidative capacity of the ETS. Addition of the ADP scavenging ("ADP trap") enzyme system tests the coupling of the cytosolic ATPases to the mitochondria [4]. The relative drop in O_2_ flux following the addition of the ADP trap did not differ between groups (Fig 4A), suggesting that the increased diffusion distance of 20% is not enough to detect impairing connectivity with this assay. The MCT RV fibres (from treated and untreated rats) responded to the addition of creatine with increased O_2_ flux, however, relative to controls this was still ~ 20% lower in the MCT and MCT + BB RV fibres (Fig 4A). Mitochondrial creatine kinase expression is decreased in the RV of MCT animals [47], therefore, cardiomyocytes may become more reliant on ADP channelling for energy transfer within the cytosol.

We measured ROS production in the presence of high ATP, which closely mimics the *in vivo* state [4]. No significant difference in ROS production between groups was found, however, the consequence of CII activity is highlighted in Fig 1 which shows a rapid increase in the rate of ROS production following the addition of succinate (CI + CII ADP-limited OXPHOS). This is likely due to superoxide production at CI due to reverse electron transport [48]. The ADP trap decreased ROS production in all groups following the addition of phosphoenolpyruvate. Phosphoenolpyruvate may have direct ROS scavenging effects [49], and is also converted to pyruvate by endogenous PK [50]. Further addition of exogenous PK with constant phosphoenolpyruvate concentration resulted in a stepwise increase in ROS production as it scavenges ADP and inhibits OXPHOS (Fig 1). Creatine decreased ROS production further in the control and MCT + BB group suggesting that the damaging effects of mitochondrial ROS are ameliorated by creatine and pyruvate.

### Mitochondrial content and sub-cellular structural changes

There was no change in citrate synthase activity in the MCT and MCT + BB hearts relative to controls, despite a decrease in maximum O_2_ flux. Citrate synthase is often used as a proxy for mitochondrial content from healthy muscle tissue [51], however, citrate synthase does not correlate with our measurements of mitochondrial fractional area calculated from myocytes. Mitochondrial area showed a 19% decrease in fractional content in the MCT groups relative to control (Fig 5D). It is possible that expansion of the myofilaments during hypertrophy compacts mitochondria into a smaller volume, with more densely packed cristae and more concentrated citrate synthase. We suggest that citrate synthase activity is not a good marker in states of disease where overall mitochondrial function may be disconnected from citrate synthase activity, particularly when the enzyme activity may not reflect the protein expression in diseased hearts [52]. It should be noted that citrate synthase activity was obtained from all cell types within the myocardial tissue sample, as were the measurements of mitochondrial function, whereas the mitochondrial area measurements were determined from within cardiomyocytes. While no decrease in connectivity was detected in the mitochondrial respiration assay, there is clearly an increase in diffusion distances between the myofilaments and mitochondria determined by the images in Fig 5B. The consequences of the expansion of myofibrils, with no change in the mitochondrial distances, remains to be resolved (Table 3). The increase in myofilament skeleton, or pixel to edge distance, were not decreased by metoprolol treatment.

### Metoprolol as a treatment of right heart failure

Although this study did not show a beneficial effect of β_1_-AR-blocker treatment on mitochondrial function in this model of right heart failure, metoprolol treatment requires further investigation with earlier interventions. We saw body weights plateau from week 4 to week 5 post injection in MCT + BB, suggesting there could be positive effects if the treatment window was expanded. β-blocker treatment using bisoprolol has previously been shown to decrease RV fibrosis in response to monocrotaline induced-PH [19]. We did not find any differences in RV fibrosis, indexed as wheat germ agglutinin labelling (Fig 6B). However, this method does not account for differences in myocyte diameter between groups. Studies which use younger animals with earlier interventions in the MCT rat model show that β_1_-AR-blocker improves survival and decreases cardiomyocyte hypertrophy in PH [18, 19, 21]. Previous improvements in creatine kinase activities and restoration of the [CrP]/[ATP] have been seen after 8 weeks of β-AR blocker treatment in rats with LV hypertrophy [35]. The MCT rat model has a rapid onset to heart failure which may not allow time for improvements in mitochondrial function or creatine kinase expression following metoprolol treatment [21]. Reportedly, the third generation non-selective β-AR blocker carvedilol, may be more effective due to its additional antioxidant properties [18]. This is reflected in recent randomised clinical trials of patients with PH which have shown good tolerance and potential cardiac benefits after 6 months of treatment with carvedilol [53], whereas there was no improvement following 6 months of selective β_1_-AR blocker bisoprolol [54]. However, carvedilol antagonism of β_2_-AR would prevent any benefits from β_2_-AR stimulation [29, 30]. Therefore, a combination of metoprolol and a mitochondrial targeted antioxidant, such as mito-Q [55], could be more therapeutic with longer dosing time, and warrants further investigation.

## Conclusion

This study tested two hypotheses, (1) that right-heart failure caused by PH contributes to impaired mitochondrial function and ADP channeling within the heart, and (2) that metoprolol treatment could regress heart failure and ameliorate mitochondrial damage. Our study showed significant impairment in RV mitochondrial function in MCT rats due to a decrease in NADH supplied OXPHOS. This was despite there being an expansion of cardiomyocyte cross-sectional area, and an increase in fractional myofilament content (Fig 5D), suggesting a *greater* need for ATP supply. Our results therefore support the hypothesis that energy deficits contribute to the contractile dysfunction that leads to right heart failure. However, although others have reported β-blocker treatment has beneficial effects in monocrotaline-induced RV dysfunction, we found no evidence here. This raises the question whether β-blocker treatment in the continued presence of elevated afterload can prevent the progression to right ventricular failure, particularly when more mature adults are studied.

## Supporting information

S1 Table Table of weekly body weights for each rat used in the study, as shown in Fig 2, together with group mean ± SEM. The table commences at Week 0 when rats were assigned to a group, and injected with either 60 mg kg^-1^ monocrotaline or an equivalent volume of saline. CON: control, MCT: monocrotaline, MCT + BB: monocrotaline plus ß-blocker. *Note: MCT 15 was excluded from growth analysis/Fig 2 since it was culled at the end of week 4 due to significant signs of heart failure.
(PDF)

S2 Table Table of individual values and group means ± SEM for the respiration of permeabilised ventricular fibres stimulated with ADP, as shown graphically in Fig 3. (PDF)

S3 Table Table of individual values and group means ± SEM for oxygen flux and ROS production from permeabilised ventricular fibres, as shown graphically in Fig 4. (PDF)

S4 Table Table of individual values and group means ± SEM of RV myocyte cross-sectional area (μm^2^), myofilament fractional area (%), and mitochondria fractional area (%), as shown in Fig 5. (PDF)

S5 Table Table of individual values and group means ± SEM of fibrosis determined from RV tissue labelled with the extracellular matrix marker, wheat germ agglutinin, as shown in Fig 6. (PDF)

S6 Table Table of individual values and group means ± SEM of morphometric data shown in Table 1. (PDF)

S7 Table Table of individual values and group means ± SEM of oxygen fluxes under different steady state respiratory states, as shown in Table 2. (PDF)

S8 Table Table of individual values and group means ± SEM of cardiomyocyte measurements, as shown in Table 3. (PDF)
