# Supplementary material for: Mitochondrial function remains impaired in the hypertrophied right ventricle of pulmonary hypertensive rats following short duration metoprolol treatment
Source: PLoS One. 2019 Apr 9;14(4):e0214740. doi: 10.1371/journal.pone.0214740 (PMC6456253; doi:10.1371/journal.pone.0214740)
Supplement: S1 Table — The table commences at Week 0 when rats were assigned to a group, and injected with either 60 mg kg-1 monocrotaline or an equivalent volume of saline. CON: control, MCT: monocrotaline, MCT + BB: monocrotaline plus ß-blocker. *Note: MCT 15 was excluded from growth analysis/Fig 2 since it was culled at the end of week 4 due to significant signs of heart failure. (PDF) [file pone.0214740.s001.pdf]

Animal	Weight week 0 (g)	Weight week 1 (g)	Weight week 2 (g)	Weight week 3 (g)	Weight week 4 (g)	Weight week 5 (g)
CON 9	300	335	352	383	409	429
CON 10	292	370	381	421	470	480
CON 13	332	360	371	400	414	436
CON 12	319	353	364	392	404	416
CON 11	321	372	383	409	432	449
CON 14	292	325	333	362	379	379
**Mean**	**309**	**353**	**364**	**395**	**418**	**432**
**SEM**	**8**	**8**	**9**	**10**	**15**	**17**
MCT 9	333	359	385	411	421	401
MCT 10	310	335	355	374	375	364
MCT 11	313	340	371	383	375	356
MCT 12	293	327	350	364	373	341
MCT 14	319	344	349	376	392	395
*MCT 15**	316	340	347	363	348	
**Mean**	**314**	**341**	**362**	**382**	**387**	**371**
**SEM**	**6**	**5**	**7**	**8**	**9**	**12**
MCT + BB 1	316	340	345	379	374	380
MCT + BB 3	323	351	349	388	402	413
MCT + BB 2	318	333	360	381	393	398
MCT + BB 6	350	359	376	400	411	415
MCT + BB 5	320	331	351	374	391	370
MCT + BB 4	336	356	382	406	393	389
**Mean**	**327**	**345**	**361**	**388**	**394**	**394**
**SEM**	**5**	**5**	**6**	**5**	**5**	**7**
