# Supplementary material for: Mitochondrial function remains impaired in the hypertrophied right ventricle of pulmonary hypertensive rats following short duration metoprolol treatment
Source: PLoS One. 2019 Apr 9;14(4):e0214740. doi: 10.1371/journal.pone.0214740 (PMC6456253; doi:10.1371/journal.pone.0214740)
Supplement: S2 Table — (PDF) [file pone.0214740.s002.pdf]

Animal	CI Leak O2 flux (pmol s-1 mg-1)	CI OXPHOS O2 flux (pmol s-1 mg-1)	CI + CII OXPHOS O2 flux (pmol s-1 mg-1)
CON 9	45.63	103.83	240.71
CON 10	51.57	125.89	200.49
CON 13	54.38	99.68	235.24
CON 12	58.96	184.54	255.91
CON 11	41.03	91.60	206.46
CON 14	46.07	95.08	256.30
**Mean**	**50**	**117**	**233**
**SEM**	**3**	**14**	**10**
MCT10	38.23	71.01	185.96
MCT9	24.16	45.35	150.32
MCT12	28.67	49.13	191.98
MCT15	42.79	88.90	215.99
MCT14	27.71	47.32	211.28
**Mean**	**32**	**60**	**191**
**SEM**	**4**	**9**	**12**
MCT + BB 1	40.30	86.33	189.23
MCT + BB 3	23.75	32.26	127.46
MCT + BB 2	37.10	110.25	213.00
MCT + BB 6	25.26	40.83	171.88
MCT + BB 5	25.65	53.27	199.72
MCT + BB 4	40.66	72.03	195.84
**Mean**	**32**	**66**	**183**
**SEM**	**3**	**12**	**12**
