# Supplementary material for: Mitochondrial function remains impaired in the hypertrophied right ventricle of pulmonary hypertensive rats following short duration metoprolol treatment
Source: PLoS One. 2019 Apr 9;14(4):e0214740. doi: 10.1371/journal.pone.0214740 (PMC6456253; doi:10.1371/journal.pone.0214740)
Supplement: S3 Table — (PDF) [file pone.0214740.s003.pdf]

Animal	CI Leak O2 flux (pmol s-1 mg-1)	CI ADP-limited OXPHOS O2 flux (pmol s-1 mg-1)	CI + CII ADP-limited OXPHOS O2 flux (pmol s-1 mg-1)	ADP trap O2 flux (pmol s-1 mg-1)	Creatine O2 flux (pmol s-1 mg-1)
CON 9	27.23	83.68	198.31	162.98	187.06
CON 10	22.62	65.33	157.42	127.36	160.74
CON 13	8.92	59.80	136.36	116.89	210.92
CON 12	14.76	58.46	235.32	177.55	232.86
CON 11	10.50	40.70	184.30	139.40	203.99
CON 14	3.72	48.62	219.19	179.42	195.42
**Mean**	**15**	**59**	**188**	**151**	**199**
**SEM**	**4**	**6**	**15**	**11**	**10**
MCT 10	19.64	44.29	136.95	107.47	143.24
MCT 9	21.31	61.78	179.73	133.32	164.63
MCT 12	16.60	52.96	133.78	115.69	141.37
MCT 15	3.48	46.02	133.08	111.39	146.64
MCT 14	12.14	54.53	160.00	127.32	182.99
**Mean**	**15**	**52**	**149**	**119**	**156**
**SEM**	**3**	**3**	**9**	**5**	**8**
MCT + BB 1	15.76	36.31	140.99	121.40	145.75
MCT + BB 3	18.97	52.58	135.18	111.23	145.80
MCT + BB 2	6.27	57.10	162.81	139.31	199.06
MCT + BB 6	12.05	46.84	146.41	103.87	122.71
MCT + BB 5	0.46	46.37	141.57	106.48	127.08
MCT + BB 4	2.99	29.51	139.64	96.01	121.75
**Mean**	**9**	**45**	**144**	**113**	**144**
**SEM**	**3**	**4**	**4**	**6**	**12**

Animal	CI Leak ROS production (pmol s-1 mg-1)	CI ADP-limited OXPHOS ROS production (pmol s-1 mg-1)	CI + CII ADP-limited OXPHOS ROS production (pmol s-1 mg-1)	ADP trap ROS production (pmol s-1 mg-1)	Creatine ROS production (pmol s-1 mg-1)
CON 9	0.077	0.055	0.304	0.367	0.125
CON 10	0.094	0.099	0.299	0.296	0.301
CON 13	0.012	0.024	0.180	0.316	0.111
CON 12	0.030	0.030	0.304	0.283	0.087
CON 11	0.028	0.042	0.378	0.244	0.063
CON 14	0.042	0.023	0.162	0.120	0.087
**Mean**	**0.047**	**0.045**	**0.271**	**0.271**	**0.129**
**SEM**	**0.013**	**0.012**	**0.034**	**0.035**	**0.035**
MCT 10	0.089	0.081	0.648	0.451	0.178
MCT 9	0.044	0.049	0.663	0.123	0.114
MCT 12	0.049	0.044	0.172	0.171	0.121
MCT 15	0.009	0.021	0.248	0.145	0.030
MCT 14	0.030	0.029	0.295	0.130	0.087
**Mean**	**0.044**	**0.045**	**0.405**	**0.204**	**0.106**
**SEM**	**0.013**	**0.010**	**0.104**	**0.062**	**0.024**
MCT + BB 1	0.041	0.033	0.267	0.161	0.071
MCT + BB 3	0.068	0.079	0.849	0.568	0.196
MCT + BB 2	0.031	0.028	0.747	0.481	0.053
MCT + BB 6	0.011	0.011	0.313	0.161	0.052
MCT + BB 5	0.040	0.065	0.340	0.221	0.089
**Mean**	**0.038**	**0.043**	**0.503**	**0.319**	**0.092**
**SEM**	**0.009**	**0.013**	**0.122**	**0.086**	**0.027**
