# Supplementary material for: Mitochondrial function remains impaired in the hypertrophied right ventricle of pulmonary hypertensive rats following short duration metoprolol treatment
Source: PLoS One. 2019 Apr 9;14(4):e0214740. doi: 10.1371/journal.pone.0214740 (PMC6456253; doi:10.1371/journal.pone.0214740)
Supplement: S4 Table — (PDF) [file pone.0214740.s004.pdf]

Animal-cell	Cross-sectional area (um2)	Myofilament fractional area (%)	Mitochondria fractional area (%)
CON13-Cell 1	**347**	42.6	41.4
CON13-Cell 2	278	35.7	43.5
CON13-Cell 3	188	40.9	42
CON13-Cell 4	282	40.6	46.4
CON13-Cell 5	183	40.4	47.3
CON13-Cell 6	362	38.6	41.4
CON13-Cell 7	60	39.1	44.1
CON14-Cell 1	165	42.7	36.1
CON14-Cell 2	177	41	44.6
CON14-Cell 3	252	31.5	42.7
CON14-Cell 4	188	37.1	45.5
CON14-Cell 5	189	41.7	42
CON14-Cell 6	112	35.5	44.6
CON14-Cell 7	301	36.7	43.8
CON14-Cell 8	163	39.6	43.5
CON12-Cell 1	359	43.8	28.7
CON12-Cell 2	226	41.7	31.5
CON12-Cell 3	355	46.2	41.7
CON12-Cell 4	215	43.4	32.6
CON12-Cell 5	249	42.6	38.5
CON12-Cell 6	227	39.9	43.9
CON12-Cell 7	303	42.3	35.6
**MEAN**	**235.5**	**40.2**	**41.0**
**SEM**	**17.5**	**0.7**	**1.1**

MCT11-Cell 1	433	36.0	34.7
MCT11-Cell 2	580	44.1	24.7
MCT11-Cell 3	173	48.4	28.2
MCT11-Cell 4	541	53.7	36.2
MCT11-Cell 5	194	51.9	36.7
MCT11-Cell 6	233	44.8	40.0
MCT11-Cell 7	659	51.5	34.4
MCT14-Cell 1	962	47.7	26.4
MCT14-Cell 2	749	45.1	29.5
MCT14-Cell 3	680	50.3	33.0
MCT14-Cell 4	775	47.7	31.5
MCT14-Cell 5	517	43.2	35.8
MCT14-Cell 6	946	48.1	36.5
MCT14-Cell 7	623	48.5	37.2
MCT15-Cell 1	542	48.0	37.0
MCT15-Cell 2	383	50.0	46.0
MCT15-Cell 3	485	30.0	32.0
MCT15-Cell 4	313	44.0	36.6
MCT15-Cell 5	272	50.7	24.0
**MEAN**	**529.5**	**46.5**	**33.7**
**SEM**	**53.9**	**1.3**	**1.3**

MCT + BB 4-Cell 1	1108	42.6	26.2
MCT + BB 4-Cell 2	872	50.8	26.3
MCT + BB 4-Cell 3	809	45.0	27.4
MCT + BB 4-Cell 4	767	47.5	34.6
MCT + BB 4-Cell 5	859	46.1	23.0
MCT + BB 4-Cell 6	1326	50.5	38.4
MCT + BB 6-Cell 1	453	53.4	38.1
MCT + BB 6-Cell 2	472	48.3	33.4
MCT + BB 6-Cell 3	786	40.9	37.2
MCT + BB 6-Cell 4	480	43.6	34.5
MCT + BB 6-Cell 5	334	40.7	31.0
MCT + BB 6-Cell 6	480	42.0	34.6
MCT + BB 6-Cell 7	334	41.8	33.8
MCT + BB 2-Cell 1	277	45.1	37.9
MCT + BB 2-Cell 2	263	49.2	32.9
MCT + BB 2-Cell 3	328	49.2	38.2
MCT + BB 2-Cell 4	213	55.8	36.0
MCT + BB 2-Cell 5	115	42.5	40.2
MCT + BB 2-Cell 6	162	37.9	36.9
**MEAN**	**549.4**	**45.9**	**33.7**
**SEM**	**77.6**	**1.1**	**1.1**
