# Supplementary material for: Mitochondrial function remains impaired in the hypertrophied right ventricle of pulmonary hypertensive rats following short duration metoprolol treatment
Source: PLoS One. 2019 Apr 9;14(4):e0214740. doi: 10.1371/journal.pone.0214740 (PMC6456253; doi:10.1371/journal.pone.0214740)
Supplement: S5 Table — (PDF) [file pone.0214740.s005.pdf]

Animal	Fibrosis (%)
CON 11	23.2
CON 13	18.3
CON 12	12.3
**Mean**	**18.0**
**SEM**	**3.1**
MCT 11	34.2
MCT 15	38.6
MCT 14	22.6
**Mean**	**31.8**
**SEM**	**4.8**
MCT + BB 2	25.1
MCT + BB 6	26.3
MCT + BB 4	29.7
**Mean**	**27.0**
**SEM**	**1.4**
