# Supplementary material for: Mitochondrial function remains impaired in the hypertrophied right ventricle of pulmonary hypertensive rats following short duration metoprolol treatment
Source: PLoS One. 2019 Apr 9;14(4):e0214740. doi: 10.1371/journal.pone.0214740 (PMC6456253; doi:10.1371/journal.pone.0214740)
Supplement: S6 Table — (PDF) [file pone.0214740.s006.pdf]

Animal No	Days post injection		Final body weight (g)	Heart weight (g)	Tibia length (mm)	Heart/tibia length (g/cm)	Lung weight (g)	Lung/tibia length (g/cm)	Liver weight (wet)	RV free wall (mm)	LV free wall (mm)
CON 9	38		436	1.56	53	0.30	2.33	0.44	15.1	1.8	3.8
CON 10	33		480	1.73	55	0.31	1.72	0.31	15.1	1.3	4.0
CON 11	41		448	1.49	56	0.27	1.56	0.28	15.9	1.2	3.8
CON 12	39		424	1.31	55	0.24	1.70	0.31	14.2	1.3	4.0
CON 13	38		462	1.51	57	0.26	1.93	0.34	15.5	1.8	4.6
CON 14	35		395	1.31	56	0.23	1.68	0.30	13.8	1.4	4.0
**Median**	**38**	**Mean**	**441**	**1.49**	**55**	**0.27**	**1.82**	**0.33**	**14.9**	**1.5**	**4.0**
**± Range**	**4**	**SEM**	**12**	**0.07**	**1**	**0.01**	**0.11**	**0.02**	**0.3**	**0.1**	**0.1**
MCT 9	36		401	2.46	56	0.44	2.25	0.40	15.3	2.4	4.3
MCT 10	35		364	2.43	54	0.45	2.07	0.39	11.5	3.3	4.2
MCT 11	34		353	1.30			1.85		12.5	3.0	3.5
MCT 12	32		341	2.03	52	0.39	2.02	0.39	13.2	3.0	3.3
MCT 14	41		393	2.08	54	0.39	2.81	0.52	12.7	1.8	4.0
MCT 15	28		348	1.85	54	0.34	3.47	0.64		2.5	3.0
**Median**	**35**	**Mean**	**367**	**2.02**	**54**	**0.40**	**2.41**	**0.47**	**13.0**	**2.7**	**3.7**
**± Range**	**7**	**SEM**	**10**	**0.17**	**1**	**0.02**	**0.25**	**0.05**	**0.6**	**0.2**	**0.2**
MCT + BB 1	31		380	1.97	52	0.38	2.28	0.44	13.9	2.5	4.0
MCT + BB 2	34		416	2.055	52	0.40	2.22	0.43	19.7	2.5	4.7
MCT + BB 3	32		398	2.24	53	0.42	2.39	0.45	16.1	2.5	4.8
MCT + BB 4	32		419	1.87	55	0.34	2.01	0.37	13.3	1.9	3.4
MCT + BB 5	35		370	1.96	54	0.36	3.3	0.61	11.1	1.9	3.8
MCT + BB 6	29		389	1.91	41	0.47	3.53	0.86	13.0	2.0	4.1
**Median**	**32**	**Mean**	**395**	**2.00**	**51**	**0.39**	**2.62**	**0.53**	**14.5**	**2.2**	**4.1**
**± Range**	**3**	**SEM**	**8**	**0.05**	**2**	**0.02**	**0.26**	**0.07**	**1.2**	**0.1**	**0.2**
