# Supplementary material for: Mitochondrial function remains impaired in the hypertrophied right ventricle of pulmonary hypertensive rats following short duration metoprolol treatment
Source: PLoS One. 2019 Apr 9;14(4):e0214740. doi: 10.1371/journal.pone.0214740 (PMC6456253; doi:10.1371/journal.pone.0214740)
Supplement: S7 Table — (PDF) [file pone.0214740.s007.pdf]

Animal	CI + CII OXPHOS (ADP) (pmols s^{-1} mg^{-1})	CI / CI + CII OXPHOS (ADP)	CI + CII ADP-limited OXPHOS (ATP) (pmols s^{-1} mg^{-1})
CON9	240.7	0.435	196.5
CON10	200.5	0.627	228.6
CON13	235.2	0.424	177.9
CON12	255.9	0.724	220.9
CON11	206.5	0.448	189.5
CON14	256.3	0.370	231.4
**Mean**	**232.5**	**0.505**	**207.4**
**SEM**	**9.8**	**0.057**	**9.2**
MCT10	186.0	0.356	275.1
MCT9	150.3	0.377	172.0
MCT12	192.0	0.343	205.3
MCT15	216.0	0.271	124.2
MCT14	211.3	0.353	214.0
**Mean**	**191.1**	**0.340**	**198.1**
**SEM**	**11.7**	**0.018**	**24.9**
MCT + BB 1	189.2	0.252	158.5
MCT + BB 3	127.5	0.298	129.9
MCT + BB 2	213.0	0.467	145.6
MCT + BB 6	171.9	0.364	133.8
MCT + BB 5	199.7	0.377	159.6
MCT + BB 4	195.8	0.367	119.1
**Mean**	**182.9**	**0.354**	**141.1**
**SEM**	**12.4**	**0.030**	**6.7**

**CI / CI + CII ADP-limited OXPHOS (ATP)**	**ET (pmols s^{-1} mg^{-1})**
0.578	308.7
0.620	210.9
0.620	288.5
0.613	305.5
0.569	317.6
0.567	332.3
**0.594**	293.9
**0.011**	17.6
0.596	
0.451	172.4
0.525	280.1
0.581	208.3
0.456	309.2
**0.522**	**242.5**
**0.030**	**31.6**
0.527	219.0
0.498	153.5
0.491	261.1
0.617	159.5
0.538	225.6
0.596	178.4
**0.544**	**199.5**
**0.021**	**17.3**
