# Supplementary material for: Mitochondrial function remains impaired in the hypertrophied right ventricle of pulmonary hypertensive rats following short duration metoprolol treatment
Source: PLoS One. 2019 Apr 9;14(4):e0214740. doi: 10.1371/journal.pone.0214740 (PMC6456253; doi:10.1371/journal.pone.0214740)
Supplement: S8 Table — (PDF) [file pone.0214740.s008.pdf]

Animal-cell	Myofilament perimeter/area	Mitochondrial perimeter/area	Myofilament skeleton to edge (nm)	Myofilament pixel to edge (nm)	Mitochondria skeleton to edge (nm)	Mitochondria pixel to edge (nm)	Mitochondria/ myofilament area
CON13-Cell 1	2.49	3.33	381.39	276.24	288.23	224.54	0.97
CON13-Cell 2	3.13	3.35	317.17	227.35	279.71	214.21	1.22
CON13-Cell 3	3.30	3.39	295.81	208.66	299.55	235.93	1.03
CON13-Cell 4	3.67	3.53	271.36	201.38	266.97	200.22	1.14
CON13-Cell 5	3.42	3.58	290.89	209.06	274.42	217.74	1.17
CON13-Cell 6	3.37	4.32	298.63	216.60	227.27	171.84	1.07
CON13-Cell 7	3.00	4.08	273.90	192.17	245.16	188.03	1.13
CON14-Cell 1	3.46	5.30	272.80	193.56	194.36	151.29	0.85
CON14-Cell 2	3.54	3.87	274.67	204.75	258.02	207.83	1.09
CON14-Cell 3	3.65	3.92	278.71	204.97	242.65	189.61	1.36
CON14-Cell 4	4.29	4.15	234.47	171.48	231.74	176.97	1.23
CON14-Cell 5	3.67	4.70	267.20	191.62	208.80	168.53	1.01
CON14-Cell 6	4.73	4.54	213.13	161.06	213.52	167.42	1.26
CON14-Cell 7	3.83	3.83	263.80	195.41	252.21	192.81	1.19
CON14-Cell 8	3.94	4.25	254.19	181.92	231.54	186.40	1.10
CON12-Cell 1	2.55	4.41	383.22	268.59	236.60	200.89	0.66
CON12-Cell 2	3.13	4.79	324.85	230.81	218.15	180.88	0.76
CON12-Cell 3	2.49	3.60			226.41	184.23	0.90
CON12-Cell 4	2.80	5.31	401.80	289.30	193.84	166.66	0.75
CON12-Cell 5	3.13	4.56	307.78	227.97	218.58	178.74	0.90
CON12-Cell 6	2.99	3.75	324.10	231.72	267.40	221.93	1.10
CON12-Cell 7	2.50	4.30	375.75	279.78	237.64	200.47	0.84
**MEAN**	**3.32**	**4.13**	**300.27**	**217.35**	**241.49**	**192.14**	**1.03**
**SEM**	**0.13**	**0.12**	**11.02**	**7.77**	**6.22**	**4.65**	**0.04**
MCT11-Cell 1	4.03	4.98	250.31	182.52	205.21	164.86	0.96
MCT11-Cell 2	2.87	6.08	348.36	254.38	177.37	147.24	0.56
MCT11-Cell 3	4.25	7.41	225.61	173.41	146.83	118.98	0.58
MCT11-Cell 4	1.99	3.49	468.39	339.15	282.40	223.31	0.67

MCT11-Cell 5	2.81	4.52	334.40	240.74	224.82	179.96	0.71
MCT11-Cell 6	3.01	4.41	325.46	245.55	223.59	179.91	0.89
MCT11-Cell 7	2.12	3.46	430.38	318.90	288.03	228.51	0.67
MCT14-Cell 1	2.46	5.21	382.68	280.94	199.90	169.74	0.55
MCT14-Cell 2	2.12	4.17	448.29	331.15	251.57	232.28	0.65
MCT14-Cell 3	2.15	4.09	429.75	304.62	254.81	219.62	0.66
MCT14-Cell 4	2.39	4.80	401.56	289.76	213.77	180.05	0.66
MCT14-Cell 5	2.94	4.18	332.52	254.15	241.91	202.00	0.83
MCT14-Cell 6	2.31	3.51	411.81	295.52	285.28	243.02	0.76
MCT14-Cell 7	2.80	4.00	342.29	248.68	246.43	200.70	0.77
MCT15-Cell 1	2.30	3.85	411.14	297.21	208.66	176.10	0.77
MCT15-Cell 2	2.63	3.46	359.18	269.89	283.21	226.01	0.92
MCT15-Cell 3	3.21	4.23	313.03	256.93	245.19	207.34	1.07
MCT15-Cell 4	3.06	4.36	325.72	249.30	249.84	217.15	0.83
MCT15-Cell 5	2.35	5.42	400.20	297.96	196.33	168.89	0.47
**MEAN**	**2.73**	**4.51**	**365.32**	**270.04**	**232.90**	**193.98**	**0.74**
**SEM**	**0.14**	**0.23**	**14.76**	**10.08**	**8.90**	**7.50**	**0.04**
MCT + BB 4-Cell 1	2.27	3.97	435.04	316.59	255.77	216.29	0.62
MCT + BB 4-Cell 2	2.01	4.56	467.30	358.68	226.11	189.78	0.52
MCT + BB 4-Cell 3	2.16	4.01	447.82	321.23	251.02	210.38	0.61
MCT + BB 4-Cell 4	2.07	3.64	466.21	334.00	266.91	218.91	0.73
MCT + BB 4-Cell 5	2.30	0.84	418.49	305.21	205.49	175.05	0.50
MCT + BB 4-Cell 6	1.52	2.74	617.64	439.04	347.89	287.56	0.76
MCT + BB 6-Cell 1	2.18	3.55	452.14	338.36	286.63	207.71	0.71
MCT + BB 6-Cell 2	2.46	3.58	388.16	272.28	299.41	225.15	0.69
MCT + BB 6-Cell 3	2.80	3.45	351.74	252.77	286.09	226.91	0.91
MCT + BB 6-Cell 4	2.76	3.92	354.51	268.54	254.95	195.50	0.79
MCT + BB 6-Cell 5	2.97	4.09	325.28	239.84	253.70	210.85	0.76
MCT + BB 6-Cell 6	2.61	3.60	369.03	270.85	279.11	218.11	0.82
MCT + BB 6-Cell 7	2.82	3.36	343.58	258.47	297.06	233.94	0.81
MCT + BB 2-Cell 1	2.45	4.34	393.61	292.56	229.68	184.98	0.84

MCT + BB 2-Cell 2	2.62	5.20	357.54	280.23	201.36	164.86	0.67
MCT + BB 2-Cell 3	2.21	4.16	431.36	331.36	237.01	206.02	0.78
MCT + BB 2-Cell 4	2.45	4.64	379.81	282.22	219.98	188.51	0.65
MCT + BB 2-Cell 5	3.92	4.98	250.98	181.64	203.88	163.52	0.95
MCT + BB 2-Cell 6	3.97	4.72	253.40	185.70	212.77	168.29	0.97
**MEAN**	**2.56**	**3.86**	**394.93**	**291.03**	**253.41**	**204.86**	**0.74**
**SEM**	**0.14**	**0.22**	**19.01**	**13.64**	**9.01**	**6.77**	**0.03**
